# Organisational climate as a social-organisational context for teacher burnout: the mediating role of attitudes towards the teaching profession

**DOI:** 10.3389/fpsyg.2026.1950351

**Published:** 2026-09-17

**Authors:** Mehmet Yaşar Kılıç, Çiler Demirtay

**Affiliations:** Department of Educational Administration, Cyprus Aydin University, Kyrenia, Cyprus

**Keywords:** attitudes towards the teaching profession, organisational climate, professional well-being, social-psychological processes, teacher burnout, teacher motivation, teacher well-being

## Abstract

**Introduction:**

Teacher burnout is an important occupational well-being concern that may reflect school-level organisational conditions and teachers’ profession-specific psychological evaluations. This study examined the direct and indirect associations among organisational climate, attitudes towards the teaching profession, and burnout among 424 lower-secondary and high-school teachers in the Turkish Republic of Northern Cyprus.

**Methods:**

A cross-sectional correlational design was employed. Data were collected using self-report measures and analysed through partial least squares structural equation modelling while controlling for age, educational qualification, tenure at the current school, and total teaching experience.

**Results:**

A more positive perception of organisational climate was associated with more favourable attitudes towards the teaching profession and lower burnout. More favourable professional attitudes were also associated with lower burnout and partially accounted for the association between organisational climate and burnout. The structural model explained 25.2% of the variance in attitudes towards the teaching profession and 41.0% of the variance in burnout. The statistically significant direct and indirect associations supported a complementary partial mediation pattern, indicating that professional attitudes represented one explanatory pathway rather than a complete explanation of the relationship between organisational climate and burnout. The model also demonstrated satisfactory out-of-sample predictive performance. When background and career-related covariates were included, the principal associations remained statistically significant.

**Discussion:**

The generally small effect sizes of the covariates indicated that teacher well-being should be considered alongside the social and organisational conditions of schools rather than primarily through demographic characteristics. The findings support a social-psychological framework in which organisational conditions, professional meaning, and occupational well-being are considered jointly. School-level practices intended to support teachers may therefore prioritise supportive leadership, mutual trust, collegial collaboration, teacher participation in decision-making, professional recognition, and working conditions that reinforce the perceived meaning and value of teaching.

## 1 Introduction

Teachers’ occupational well-being plays an important role in maintaining the quality of educational processes and the effective functioning of schools (Agyapong et al., 2022; Madigan and Kim, 2021). Teaching, however, involves a range of occupational demands, including intensive interpersonal interaction, emotional labour, time pressure, student needs, and administrative responsibilities (Agyapong et al., 2022; Mijakoski et al., 2022). When these demands remain insufficiently balanced by adequate resources over time, they may be associated with poorer psychological and occupational well-being and a greater risk of burnout (Bakker and Demerouti, 2017; Maslach and Leiter, 2016). Burnout is defined as an occupational phenomenon associated with chronic workplace stress that has not been successfully managed and is characterised by depleted energy, mental distance from work, and reduced professional efficacy (Maslach and Leiter, 2016; World Health Organization, 2019). Its associations with lower job satisfaction and intentions to leave the profession indicate that teacher burnout is not only an individual psychological concern but also an important occupational well-being issue for educational organisations (Madigan and Kim, 2021).

Teacher burnout cannot be explained solely by individual characteristics or personal coping capacities (Agyapong et al., 2022; Mijakoski et al., 2022). Workload, insufficient administrator support, organisational conflict, adverse collegial relationships, and unfavourable working conditions have been associated with teachers’ occupational strain (Agyapong et al., 2022; Mijakoski et al., 2022). Teachers’ psychological and professional experiences should therefore not be considered independently of the social and organisational characteristics of the schools in which they work (Schneider et al., 2013; Toropova et al., 2021). Organisational climate refers to employees’ shared or similar perceptions of organisational policies, practices, management processes, and interpersonal relationships (Schneider et al., 2013). In schools, organisational climate is formed through features that shape teachers’ everyday work experiences, including administrator behaviour, trust, communication, support, collegial relationships, and collaboration (Hoy and Tarter, 1997; Yılmaz and Altınkurt, 2013). Organisational climate may therefore be regarded as a central contextual construct for understanding the social and organisational correlates of teacher burnout (Malinen and Savolainen, 2016; Mijakoski et al., 2022).

The association between organisational climate and teacher burnout can be interpreted with reference to Job Demands-Resources theory (Bakker and Demerouti, 2017). In the present study, JD-R theory is used as an interpretative framework rather than as a comprehensive empirical test of the full JD-R model. According to the theory, job demands require sustained physical, cognitive, or emotional effort, whereas job resources facilitate goal attainment, limit the adverse consequences of demands, and support employee motivation (Bakker and Demerouti, 2017). Specific job demands such as workload, time pressure, role conflict, or emotional demands were not measured separately in this study; consequently, the model cannot determine which particular demands are associated with burnout or compare their relative importance. Instead, JD-R theory is used to clarify why the social and organisational conditions represented in teachers’ climate perceptions may be relevant to burnout. Organisational characteristics such as administrator support, trust, collegial collaboration, autonomy, and participation can function as job resources that may help teachers manage occupational demands (Granziera et al., 2021; Skaalvik and Skaalvik, 2018). Organisational climate itself, however, is not treated as a single job resource. Rather, it is conceptualised as a higher-order perceptual context reflecting teachers’ overall experience of resource-relevant social and organisational conditions within the school. The association between organisational conditions and burnout may also extend beyond a direct pathway, because teachers’ experiences within the school environment may be connected to their evaluations of the meaning and value of their profession (Toropova et al., 2021; van Beurden et al., 2017).

Attitudes towards the teaching profession are conceptualised in the present study as a profession-specific evaluative orientation: the cognitive and affective appraisals through which teaching itself is regarded as an attitude object (Çetin, 2006; Pekmezci and Dağdeviren Ertaş, 2024). Operationally, this orientation is represented by three related facets—Love, Value, and Adaptation. Love reflects affective attraction to and positive feelings about teaching; Value reflects the perceived worth and importance assigned to the profession; and Adaptation reflects perceived fit with, and acceptance of, the requirements of the teaching role. This referent-based definition is important for distinguishing professional attitudes from the other constructs in the model. Organisational climate concerns teachers’ perceptions of the social, managerial, and relational context of the school in which they work (Schneider et al., 2013), whereas burnout concerns experienced occupational strain expressed through emotional exhaustion, depersonalisation, and reduced personal accomplishment (Maslach and Jackson, 1981). Thus, although unfavourable professional attitudes may be associated with burnout, ATTP is intended to capture how teaching as a profession is cognitively and affectively evaluated, whereas burnout reflects an occupational state characterised by exhaustion, psychological distancing, and reduced professional efficacy (Pekmezci and Dağdeviren Ertaş, 2024; Maslach and Leiter, 2016).

Previous research has examined the associations between organisational conditions and teacher burnout through psychological and professional mechanisms such as self-efficacy, job satisfaction, organisational belonging, professional identity, and teaching motivation (Malinen and Savolainen, 2016; Skaalvik and Skaalvik, 2011). However, the explanatory role of attitudes towards the teaching profession in the indirect association between organisational climate and burnout has received limited attention (Dikmen et al., 2023; Sezer and Çevik, 2023). Organisational climate represents teachers’ perceptions of their social and organisational work environment, whereas attitudes towards the teaching profession represent their cognitive and affective orientations towards teaching. Examining these constructs within the same model may therefore contribute to a more comprehensive understanding of the organisational and psychological foundations of teacher burnout (Bakker and Demerouti, 2017; Schneider et al., 2013).

### 1.1 Theoretical background and hypothesis development

#### 1.1.1 Teacher well-being and burnout in educational organisations

Teacher well-being is a multidimensional concept referring to the overall quality of teachers’ psychological, emotional, and professional experiences while performing their occupational roles (Granziera et al., 2021; Skaalvik and Skaalvik, 2018). It is not limited to the absence of adverse states such as stress, anxiety, or burnout, but also encompasses positive experiences such as professional satisfaction, a sense of meaning, positive affect, and sustained functioning (Granziera et al., 2021). Teachers’ well-being is important for their capacity to maintain effective teaching, fulfil professional responsibilities, and remain engaged with their teaching roles (Skaalvik and Skaalvik, 2018). Teacher well-being should therefore be considered not only in relation to individual quality of life but also in relation to the continuity and quality of educational processes (Agyapong et al., 2022; Madigan and Kim, 2021).

Burnout is one of the principal forms of occupational strain that may threaten teachers’ professional well-being (Maslach and Leiter, 2016). The World Health Organization defines burnout as an occupational phenomenon associated with chronic workplace stress that has not been successfully managed; it is characterised by depleted energy, mental distance from work, and reduced professional efficacy (World Health Organization, 2019). The intensive interpersonal interaction, emotional responsibility, time pressure, changing student needs, and multiple task demands involved in teaching may expose teachers to prolonged occupational strain (Agyapong et al., 2022; Mijakoski et al., 2022). When these demands persist without being adequately managed, teachers may experience reduced physical and emotional energy, greater psychological distance from their professional roles, and weaker perceptions of professional efficacy (Maslach and Leiter, 2016).

The consequences of teacher burnout extend beyond individual fatigue or temporary work stress (Agyapong et al., 2022). Burnout has been associated with lower job satisfaction, reduced professional motivation, and stronger intentions to leave teaching (Madigan and Kim, 2021; Skaalvik and Skaalvik, 2011). It is therefore regarded as an important risk to teachers’ psychological health and professional functioning (Maslach and Leiter, 2016). At the same time, burnout among teachers should not be reduced to personal characteristics or individual coping capacities; the conditions under which teaching is performed also need to be considered (Agyapong et al., 2022; Mijakoski et al., 2022). Understanding teacher burnout and supporting occupational well-being consequently require attention to both individual experiences and the social and organisational characteristics of educational settings.

#### 1.1.2 Organisational climate as a perceived social-organisational context

Organisational climate is a multidimensional construct referring to employees’ shared or similar perceptions of organisational policies, practices, working conditions, and interpersonal relationships. It arises not only from an organisation’s formal characteristics but also from the meanings employees assign to their everyday work experiences (Schneider et al., 2013). In schools, organisational climate encompasses interactions between administrators and teachers, management practices, and professional relationships among teachers (Hoy and Tarter, 1997; Yılmaz and Altınkurt, 2013). Supportive, directive, and restrictive administrator behaviours represent its managerial aspects, whereas collegiality, intimacy, and disengagement reflect teacher-related aspects (Hoy and Tarter, 1997; Yılmaz and Altınkurt, 2013). School organisational climate therefore extends beyond the physical work environment to represent the overall quality of the social, managerial, and professional context in which teachers perform their roles.

Organisational climate should not be equated with formal arrangements or organisational processes themselves. Leadership practices, rules, communication structures, decision-making arrangements, and other school processes occur at the organisational level, whereas climate refers to how such conditions, practices, and relationships are perceived and experienced by organisational members (Schneider et al., 2013). This distinction is consistent with research in which school climate is treated as a psychosocial context that can be examined separately from specific professional or organisational processes, such as reflective culture (Brestovanský and Sopoliga, 2026). Teachers and administrators within the same school may therefore evaluate organisational trust, rules, support, and encouragement for new initiatives differently (Veletić et al., 2023). Organisational climate consequently does not consist solely of objective working conditions; it is shaped through members’ interpretations and shared sense-making of their everyday organisational experiences (Schneider et al., 2013; Veletić et al., 2023). Trust, psychological safety, and regular professional collaboration among teachers are particularly important features of a positive school climate (Hammar Chiriac et al., 2024). Teachers may therefore be viewed not only as recipients of organisational conditions but also as participants in the social processes through which climate perceptions are formed and maintained.

From the perspective of Job Demands-Resources theory, supportive leadership, open communication, trust, collaboration, autonomy, and participation in decision-making may represent resource-relevant social and organisational conditions that facilitate teachers’ professional work (Bakker and Demerouti, 2017). In the present study, however, these conditions were not operationalised as separate job-resource variables, and their relative contributions were therefore not estimated. Instead, the Organisational Climate Scale captures teachers’ broader perceptions of the school’s managerial, collegial, and professional environment through its six dimensions (Hoy and Tarter, 1997; Yılmaz and Altınkurt, 2013). Accordingly, organisational climate is conceptualised neither as a single job resource nor as an objective sum of resources, but as a higher-order perceptual construct reflecting the overall quality of the social and organisational context as experienced by teachers. A climate characterised by supportive administrator behaviour, collegial relationships, trust, and mutual support may be consistent with the perceived availability of resources, whereas restrictive management, disengagement, limited communication, and conflictual relationships may be consistent with a less supportive resource context. JD-R theory is therefore used to interpret the relevance of these resource-related organisational conditions to teachers’ professional and psychological experiences, rather than to claim that the present design identifies specific demands or resources and their relative effects.

Although a multidimensional construct may be specified formatively when its dimensions are conceived as components that jointly define the construct (Hair et al., 2019), a reflective higher-order specification was adopted in the present study because the six dimensions were conceptualised as related manifestations of teachers’ broader perceptions of the school’s social, managerial, and professional environment, rather than as independent components whose combination defines organisational climate. This interpretation is consistent with approaches to organisational climate that emphasise employees’ perceptions of interconnected organisational practices, relationships, and processes (Hoy and Tarter, 1997; Schneider et al., 2013; Yılmaz and Altınkurt, 2013). Under this conceptualisation, the dimensions were expected to share a common underlying climate perception while retaining their substantive distinctiveness. The empirical coherence of this specification was therefore examined through the relationships among the six dimensions and the higher-order measurement results.

#### 1.1.3 Organisational climate and attitudes towards the teaching profession

Attitudes towards the teaching profession refer to teachers’ cognitive, affective, and behavioural orientations towards the value, meaning, and requirements of teaching (Andronache et al., 2014; Pekmezci and Dağdeviren Ertaş, 2024). Favourable attitudes are associated with perceiving the teaching role as meaningful and valuable, accepting professional responsibilities, and willingly continuing teaching activities (Pekmezci and Dağdeviren Ertaş, 2024). Teachers’ evaluations of their profession, however, are not formed solely through individual tendencies; they may also be connected to the social and organisational environment in which teaching is carried out (Toropova et al., 2021).

A positive organisational climate reflects perceptions that social and organisational resources such as administrator support, mutual trust, open communication, collegial collaboration, autonomy, and participation in decision-making are available (Collie et al., 2012; Skaalvik and Skaalvik, 2018). Under such conditions, teachers may be more likely to feel supported, perceive their contributions as valued, and experience themselves as part of the school’s aims (Skaalvik and Skaalvik, 2011; Toropova et al., 2021). Supportive working environments may consequently be associated not only with teachers’ evaluations of their schools but also with more favourable evaluations of their professional roles (Sezer and Çevik, 2023; Toropova et al., 2021). Conversely, limited support, inadequate communication, organisational conflict, and excessive control may co-occur with less favourable evaluations of the teaching role (Skaalvik and Skaalvik, 2011).

From the perspective of Job Demands-Resources theory, organisational climate can be regarded as a contextual construct reflecting teachers’ perceptions of the overall quality of their social and organisational resources (Bakker and Demerouti, 2017). A school environment in which supportive resources are perceived to be available may be associated with teachers viewing their profession as more meaningful, valuable, and sustainable. Previous research has reported positive associations between school climate and attitudes towards the teaching profession (Sezer and Çevik, 2023). On the basis of these theoretical arguments and empirical findings, the following hypothesis was proposed:

*H1:* A more positive perceived organisational climate in schools is associated with more favourable attitudes towards the teaching profession.

#### 1.1.4 Organisational climate and teacher burnout

Teacher burnout is associated not only with the intensity of occupational demands but also with the social and organisational conditions under which those demands are experienced (Agyapong et al., 2022; Mijakoski et al., 2022). Organisational climate reflects the overall quality of teachers’ experiences of administrator support, collegial relationships, trust, communication, participation, and work organisation (Grayson and Alvarez, 2008). A school climate characterised by support, collaboration, and mutual trust may provide social and organisational resources that help teachers manage occupational demands (Malinen and Savolainen, 2016). In contrast, insufficient administrator support, conflictual relationships, limited communication, and excessive control may be associated with poorer access to resources and greater occupational strain (Grayson and Alvarez, 2008; Mijakoski et al., 2022).

Research examining organisational climate and teacher burnout has generally reported that more positive climate perceptions are associated with lower burnout. Grayson and Alvarez (2008) found that managerial and relational features of school climate were associated with different dimensions of burnout and that part of this association was accounted for by teachers’ job satisfaction. In a longitudinal study, Malinen and Savolainen (2016) reported both a direct association between perceived school climate and teacher burnout and an indirect association through teacher self-efficacy. More recent studies have likewise found that positive school climate is negatively associated with teacher burnout and is connected to teachers’ psychological experiences alongside personal and professional resources (Postigo-Zegarra et al., 2025; Yang and Zhou, 2025).

According to Job Demands-Resources theory, persistent job demands that are insufficiently balanced by resources may activate a health-impairment process associated with energy depletion and burnout (Bakker and Demerouti, 2017). Within this framework, positive organisational climate is treated not as a single job resource but as a contextual construct reflecting teachers’ perceptions of the overall availability and quality of social and organisational resources. A supportive climate may be associated with lower psychological costs of occupational demands, whereas an adverse climate may accompany greater strain. On the basis of these theoretical arguments and empirical findings, the following hypothesis was proposed:

*H2:* A more positive perceived organisational climate in schools is associated with lower teacher burnout.

#### 1.1.5 Attitudes towards the teaching profession and teacher burnout

Teachers’ cognitive and affective evaluations of their profession may shape the meaning assigned to occupational experiences and the way in which teaching roles are approached (Pekmezci and Dağdeviren Ertaş, 2024). In the present model, these evaluations are treated as conceptually related to, but distinct from, burnout. ATTP concerns the valence of teachers’ evaluation of teaching itself—whether the profession is loved, valued, and experienced as compatible with the requirements of the teaching role—whereas burnout concerns a state of occupational strain reflected in exhaustion, depersonalisation, and diminished personal accomplishment (Maslach and Jackson, 1981; Maslach and Leiter, 2016). Some attitudinal items necessarily contain affective wording because attitudes towards the teaching profession encompass cognitive and affective evaluations of the profession (Pekmezci and Dağdeviren Ertaş, 2024). However, their referent remains the profession rather than symptoms of depletion or psychological detachment characteristic of burnout (Maslach and Leiter, 2016). This distinction allows an association between professional attitudes and burnout to be hypothesised without treating the two constructs as interchangeable.

Within the motivational process proposed by Job Demands-Resources theory, favourable professional attitudes may be connected to how teachers appraise occupational demands and the extent to which they invest cognitively and emotionally in their work (Bakker and Demerouti, 2017). Regarding teaching as valuable and meaningful may be associated with maintaining professional purpose and teaching motivation, which may in turn be negatively related to psychological distancing and burnout. Unfavourable professional attitudes may instead co-occur with a weaker psychological connection to work and more adverse appraisals of occupational demands. Attitudes towards the teaching profession should not, however, be treated as a personal resource in the conventional sense; rather, they represent a psychological and motivational orientation reflecting how teachers evaluate their profession cognitively and affectively.

Empirical evidence suggests that more favourable attitudes towards the teaching profession may be associated with lower burnout. A study of practising physical education and sport teachers reported a negative and statistically significant association between professional attitudes and occupational burnout (Şen and Koçak, 2022). Studies of pre-service teachers have similarly identified inverse associations between burnout and attitudes towards teaching and have suggested that motivation to teach may play an explanatory role in this relationship (Dikmen et al., 2023). Findings from pre-service teachers, however, should not be directly generalised to practising teachers. Longitudinal research among practising teachers has shown that teaching motivation, intentions to leave the profession, and dimensions of burnout are related over time (Skaalvik and Skaalvik, 2020). On the basis of these theoretical arguments and empirical findings, the following hypothesis was proposed:

*H3:* More favourable attitudes towards the teaching profession are associated with lower teacher burnout.

#### 1.1.6 The mediating role of attitudes towards the teaching profession

Organisational climate may be related to teacher burnout both directly and indirectly through teachers’ evaluations of their profession. Supportive leadership, trust, collegial collaboration, recognition, and professional autonomy may be associated with teachers feeling valued and evaluating their teaching roles as more meaningful (Skaalvik and Skaalvik, 2018; Toropova et al., 2021). Conversely, insufficient support, conflictual relationships, excessive control, and incongruence between teachers’ professional values and school practices may co-occur with less favourable evaluations of the profession (Skaalvik and Skaalvik, 2011; van Beurden et al., 2017). Attitudes towards the teaching profession may therefore be considered a profession-specific cognitive and affective orientation that could help explain the association between organisational experiences and occupational strain.

According to Job Demands-Resources theory, social and organisational job resources may support employee motivation and psychological connection to work and may thereby be associated with a lower risk of burnout (Bakker and Demerouti, 2017). Within this framework, positive organisational climate reflects the perceived overall quality of the organisational resources available to teachers, whereas attitudes towards the teaching profession reflect the extent to which teaching is viewed as valuable, meaningful, and worth sustaining under those conditions. A supportive school climate may consequently be associated with more favourable professional attitudes, which may in turn be associated with lower burnout. This account positions attitudes towards the teaching profession not as a conventional personal resource but as a profession-specific orientation that may help explain the connection between organisational experiences and teachers’ psychological and occupational well-being.

Although research directly examining the mediating role of attitudes towards the teaching profession in this specific association remains limited, findings concerning related constructs suggest that profession-related psychological mechanisms may form part of indirect associations with teacher burnout. Associations between teachers’ professional identity and burnout have been reported through work engagement and psychological capital (Zhang et al., 2024). Similarly, work engagement and psychological capital have been identified as parallel and sequential mediators in the association between career calling and burnout (Zhao et al., 2022). Although these constructs are conceptually distinct from attitudes towards the teaching profession, they indicate that the meaning teachers assign to their profession and their professional orientations may be relevant psychological mechanisms in the explanation of burnout. Related mediation research further suggests that the association between organisational conditions and burnout may operate through multiple mechanisms. Lim and Eo (2014) identified a mediating role for collective teacher efficacy in associations between school organisational climate and burnout, Malinen and Savolainen (2016) reported an indirect association through teacher efficacy in behaviour management, and Junça-Silva and Freire (2022) identified work-family conflict as an intervening process between dimensions of organisational climate and burnout.

The mediator examined in the present study is conceptually distinct from these efficacy-based mechanisms. Teacher self-efficacy concerns teachers’ beliefs about their capability to organise and execute the actions required to perform teaching tasks, whereas collective teacher efficacy concerns shared beliefs about the faculty’s capability to influence educational outcomes. By contrast, attitudes towards the teaching profession concern teachers’ cognitive and affective evaluation of the value and meaning of teaching as a profession rather than their perceived capability to perform teaching tasks successfully. A teacher may therefore feel highly capable of teaching while holding a less favourable evaluation of the profession itself, or may value the profession strongly while experiencing doubts about personal capability. This distinction is theoretically consequential because attitudes towards the teaching profession represent an evaluation of the profession itself rather than a belief about individual or collective capability or a generic personal resource. Evidence among practising teachers has linked more favourable professional attitudes with lower burnout (Şen and Koçak, 2022), while research with pre-service teachers has connected attitudes towards teaching with teaching motivation and burnout-related processes (Dikmen et al., 2023; Pekmezci and Dağdeviren Ertaş, 2024). Together with evidence that school climate is associated with professional attitudes (Sezer and Çevik, 2023), these findings provide a theoretically ordered basis for examining professional attitude as an intervening psychological orientation between organisational climate and burnout. On the basis of the preceding theoretical arguments and empirical findings, the following hypothesis was proposed:

*H4:* Organisational climate in schools is indirectly and negatively associated with teacher burnout through attitudes towards the teaching profession.

#### 1.1.7 Background and career-related covariates

Teachers’ attitudes towards their profession and their burnout levels may be associated not only with organisational working conditions but also with background and career-related characteristics such as age, educational qualification, and professional experience (Kuyumcu and Kaya, 2020; Yorulmaz and Altınkurt, 2018). Meta-analyses and systematic reviews of teacher burnout, however, indicate that the associations of educational qualification, age, and professional experience are generally small and do not show a consistent direction across studies (Polatcan et al., 2020; Yorulmaz and Altınkurt, 2018). Studies of attitudes towards the teaching profession have likewise reported some differences by age and experience, although findings appear to vary according to sample and occupational context (Kuyumcu and Kaya, 2020; Mirze and Yılmaz, 2022). These characteristics were therefore included as covariates rather than treated as the principal explanatory constructs of the study, allowing the focal structural associations to be evaluated while accounting for relevant background variation (Becker, 2005).

Age and total teaching experience are related but represent different aspects of teachers’ professional lives (İlya, 2023; Polatcan et al., 2020). Age is connected to teachers’ broader life and career stage, whereas total teaching experience represents the length of exposure to teaching responsibilities and occupational demands (İlya, 2023). The varying findings concerning the associations of age and total experience with burnout make a single directional expectation difficult to justify (Polatcan et al., 2020; Yorulmaz and Altınkurt, 2018). Including these variables separately as covariates enables life-stage differences to be distinguished conceptually from accumulated teaching experience (Becker, 2005).

Educational qualification may be associated with teachers’ professional expectations, career opportunities, and evaluations of the teaching role, although its overall association with burnout has been reported to be very small (Yorulmaz and Altınkurt, 2018). Tenure at the current school differs from total teaching experience because it reflects the length of exposure to the management practices, social relationships, and organisational conditions of a particular school (Schneider et al., 2013; van Beurden et al., 2017). Longer tenure at one school may be associated with greater familiarity with organisational relationships and school processes, but it may also represent prolonged exposure to the same organisational conditions (Schneider et al., 2013; van Beurden et al., 2017). Age, educational qualification, tenure at the current school, and total teaching experience were therefore included as background and career-related covariates for attitudes towards the teaching profession and teacher burnout (Becker, 2005). Because previous findings differ in direction and magnitude and these characteristics are not central theoretical constructs in the present model, no directional hypotheses were formulated for their associations (Becker, 2005; Yorulmaz and Altınkurt, 2018).

#### 1.1.8 The present study

Previous research has linked teacher burnout to a range of organisational and psychological variables, including school climate, administrator support, job satisfaction, self-efficacy, professional identity, and teaching motivation (Malinen and Savolainen, 2016; Skaalvik and Skaalvik, 2011; Zhang et al., 2024). However, profession-specific psychological mechanisms that may help explain how organisational climate is associated with teacher burnout remain comparatively underexamined. In particular, the role of attitudes towards the teaching profession in the indirect association between teachers’ organisational experiences and burnout has not been sufficiently clarified (Dikmen et al., 2023; Sezer and Çevik, 2023). This gap supports examining teacher burnout not only in relation to organisational conditions but also as part of a social-psychological process connected to the meaning and value teachers assign to their profession.

The study offers three related contributions. First, organisational climate, attitudes towards the teaching profession, and burnout are integrated within the same model, allowing teachers’ social-organisational work environment and profession-specific psychological orientations to be considered together. Second, attitudes towards the teaching profession are examined as a cognitive and affective mechanism that may help account for the association between organisational climate and burnout. The model therefore tests whether organisational climate is related to burnout both directly and indirectly through teachers’ evaluations of their profession. Third, age, educational qualification, tenure at the current school, and total teaching experience are included as covariates, enabling the focal structural associations to be evaluated while accounting for background and career-related characteristics.

The theoretical contribution of the proposed model does not rest on claiming that the individual bivariate associations are themselves novel. Rather, it lies in distinguishing teachers’ appraisal of their school context from their appraisal of the teaching profession and testing whether these two levels of experience are statistically connected in relation to burnout. Previous mediation research has emphasised mechanisms such as self-efficacy (Malinen and Savolainen, 2016; Yang and Zhou, 2025), basic psychological need satisfaction (Harrison et al., 2025), emotion regulation (Li, 2025), and work engagement or psychological capital (Zhang et al., 2024; Zhao et al., 2022). By contrast, attitudes towards the teaching profession capture a profession-specific evaluation of whether teaching is valued, meaningful, and worth sustaining. Positioning this construct between organisational climate and burnout therefore extends existing process-oriented accounts by examining a distinct profession-specific evaluative pathway rather than proposing another general personal resource.

Accordingly, the primary aim of this study was to examine the direct and indirect associations among organisational climate in schools, attitudes towards the teaching profession, and teacher burnout. The associations of organisational climate with professional attitudes and burnout, as well as the association between professional attitudes and burnout, were tested. The statistical mediating role of attitudes towards the teaching profession in the association between organisational climate and teacher burnout was also evaluated. Teacher burnout was thereby examined within an integrated framework combining the social and organisational conditions of schools with teachers’ cognitive and affective orientations towards their profession.

Conducting the study among lower-secondary and high-school teachers in the TRNC provides evidence from an educational context that has been comparatively underrepresented in research on the organisational and psychological correlates of teacher well-being. In this respect, the study offers a context-sensitive examination of how the proposed associations are expressed within this educational setting. Because a cross-sectional design was used, however, the directional paths specified in the model were not interpreted as evidence of temporal order or causality. JD-R theory was used to provide an interpretative account of why resource-relevant social and organisational conditions may be associated with professional attitudes and burnout; the study was not designed as a comprehensive test of the JD-R model because specific job demands and discrete job resources were not separately operationalised. The proposed paths were therefore treated as theoretically grounded statistical associations informed by JD-R theory and prior empirical research.

## 2 Materials and methods

### 2.1 Research design

This quantitative study employed a cross-sectional correlational design (Creswell and Creswell, 2018). The direct and indirect associations among organisational climate in schools, attitudes towards the teaching profession, and teacher burnout were examined. The statistical mediating role of attitudes towards the teaching profession in the association between organisational climate and teacher burnout was also tested (Hayes, 2022). Given the cross-sectional design, the model paths were interpreted as theoretically justified patterns of association rather than causal effects. The proposed research model is presented in Figure 1.

**Figure 1:**
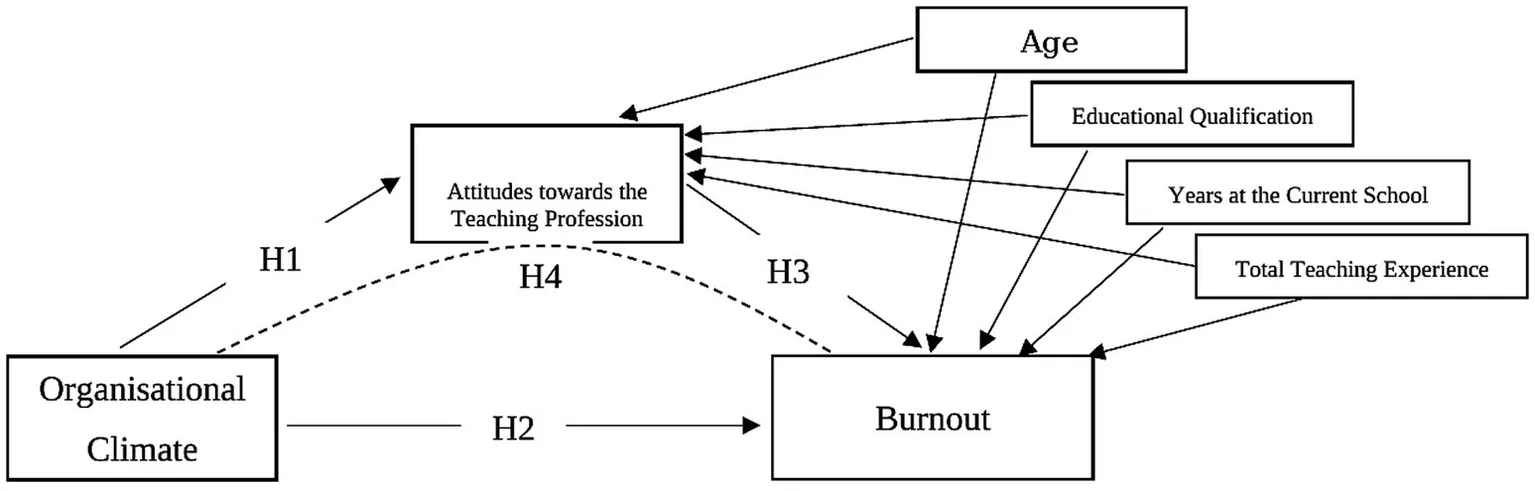
Proposed research model with background and career-related covariates.

### 2.2 Sample

The target population comprised teachers employed at the lower-secondary and high-school levels in the Turkish Republic of Northern Cyprus during the 2025–2026 academic year. According to the 2025–2026 Statistical Yearbook of the Ministry of National Education of the Turkish Republic of Northern Cyprus, 3,572 teachers were employed across these two educational levels in total (Ministry of National Education of the Turkish Republic of Northern Cyprus, 2026). Accordingly, the combined lower-secondary and high-school teacher population reported in the official statistics was used as the target-population frame for the study.

A stratified convenience-sampling procedure was employed. In the first stage, Nicosia, Famagusta, Kyrenia, Morphou, İskele, and Lefke were defined as geographical strata. This stratification was used to reduce the likelihood that participants would be concentrated in a single region and to ensure the inclusion of lower-secondary and high-school teachers working in different parts of the TRNC. Because regional teacher counts across the two educational levels were not available in a form suitable for proportional allocation, proportional sampling across the geographical strata could not be undertaken.

In the second stage, teachers were approached in accessible lower-secondary and high schools for which permission to collect data had been obtained in each region. To be eligible, participants were required to be employed as teachers at the lower-secondary or high-school level in the TRNC during the 2025–2026 academic year and to participate voluntarily. Although efforts were made to recruit participants from each geographical stratum, the number of participants from each region was not assumed to represent the proportional regional distribution of lower-secondary and high-school teachers. The sampling procedure therefore combined geographical stratification with the recruitment of accessible and voluntary participants within each stratum (Creswell and Creswell, 2018).

Because the target population was finite (*N* = 3,572), the minimum required sample size was estimated using Cochran’s initial sample-size formula followed by the finite population correction (Cochran, 1977): n₀ = (z^2^ × p × q)/d^2^; n = n₀/[1 + (n₀ − 1)/N].

In these formulas, n₀ represents the initial sample-size estimate, n represents the finite-population-adjusted sample size, N represents the population size, z represents the standard value corresponding to the selected confidence level, p represents the probability of occurrence, q represents the probability of non-occurrence, and d represents the accepted margin of sampling error. Using z = 1.96 for a 95% confidence level, p = 0.50, q = 0.50, d = 0.05, and N = 3,572 produced an initial estimate of approximately 384 and a finite-population-adjusted minimum of approximately 347 participants. Following data screening, valid responses from 424 teachers were retained for analysis, exceeding the minimum sample-size requirement.

Sample adequacy was also assessed through an a posteriori power analysis conducted using G*Power 3.1 (Faul et al., 2009). A linear multiple-regression model within the F-test family was specified. Because the most complex regression equation in the proposed model included organisational climate, attitudes towards the teaching profession, and four background and career-related covariates, six predictors were entered into the power analysis. Using a medium effect size of f^2^ = 0.15, a significance level of 0.05, and a sample size of 424, the resulting statistical power exceeded 0.99. The sample size was therefore considered adequate for examining the direct and indirect associations proposed in the research model while accounting for the included covariates. The demographic characteristics of the final sample are presented in Table 1.

**Table 1:** Demographic characteristics of study participants.

Variable	Group	Frequency (*n*)	Percentage (%)
Gender	Male	172	40.6
	Female	252	59.4
Age	20–29 years	108	25.5
	30–39 years	135	31.8
	40–49 years	97	22.9
	50 years or over	84	19.8
Highest level of education	Bachelor’s degree	225	53.1
	Master’s degree	172	40.6
	Doctoral degree	27	6.4
Professional experience	1–5 years	83	19.6
	6–10 years	82	19.3
	11–15 years	96	22.6
	16–20 years	95	22.4
	21 years or more	68	16.0
Tenure at the current school	1–5 years	155	36.6
	6–10 years	116	27.4
	11–15 years	77	18.2
	16–20 years	54	12.7
	21 years or more	22	5.2

Of the 424 participants, 172 were men (40.6%) and 252 were women (59.4%), indicating that women constituted the larger proportion of the sample. Regarding age, 108 participants were aged 20–29 years (25.5%), 135 were aged 30–39 years (31.8%), 97 were aged 40–49 years (22.9%), and 84 were aged 50 years or over (19.8%). Although teachers aged 30–39 years constituted the largest age group, participants from a broad range of age categories were represented.

In terms of educational qualification, 225 participants held a bachelor’s degree (53.1%), 172 held a master’s degree (40.6%), and 27 held a doctoral degree (6.4%). Overall, 46.9% of the participants held a postgraduate qualification, showing that teachers with master’s or doctoral degrees constituted a substantial proportion of the sample.

The educational profile of the sample was interpreted cautiously. As a broad international point of reference, TALIS 2024 reported that 57% of teachers across participating education systems held a master’s degree or equivalent, although this benchmark should not be treated as directly comparable with the present lower-secondary and high-school sample because qualification structures vary across systems and educational levels (OECD, 2025). Moreover, the available 2025–2026 TRNC statistical yearbook does not report the educational-qualification distribution specifically for lower-secondary and high-school teachers. It was therefore not possible to determine whether the 46.9% postgraduate proportion in the present sample over- or under-represented the target population. Educational qualification was consequently retained as a covariate in the structural analyses.

Regarding total teaching experience, 83 participants had 1–5 years of experience (19.6%), 82 had 6–10 years (19.3%), 96 had 11–15 years (22.6%), 95 had 16–20 years (22.4%), and 68 had 21 years or more (16.0%). The distribution included teachers from both early-career and more experienced groups.

Regarding the number of years worked at the current school, 155 participants had worked at their current school for 1–5 years (36.6%), 116 for 6–10 years (27.4%), 77 for 11–15 years (18.2%), 54 for 16–20 years (12.7%), and 22 for 21 years or more (5.2%). Overall, 63.9% of the participants had worked at their current school for no more than 10 years. The sample also included teachers with longer tenure at their current schools.

### 2.3 Measures

Data were collected using a Personal Information Form, the Organisational Climate Scale, the Attitude towards the Teaching Profession Scale, and the Maslach Burnout Inventory. Items requiring reverse scoring were recoded before the analyses in accordance with the scoring instructions for each instrument. Subdimension scores were calculated for each instrument and used as indicators of the corresponding higher-order constructs.

The Personal Information Form was developed by the researchers to obtain information about participants’ gender, age, highest completed educational qualification, total teaching experience, and tenure at their current school. Age, educational qualification, total teaching experience, and tenure at the current school were treated as ordered categorical variables. No personally identifying information was requested.

Teachers’ perceptions of organisational climate were measured using the Organisational Climate Scale developed by Hoy and Tarter (1997) and adapted into Turkish by Yılmaz and Altınkurt (2013). The scale comprises 39 items across six dimensions. Items are rated on a four-point Likert-type scale ranging from 1 (rarely occurs) to 4 (very frequently occurs). In the Turkish adaptation study, Cronbach’s alpha coefficients for the subdimensions ranged from 0.70 to 0.89 (Yılmaz and Altınkurt, 2013). Items requiring reverse scoring were recoded in accordance with the scoring instructions. Scores for the six subdimensions were calculated and used as indicators of the higher-order organisational climate construct. Higher scores indicate a more positive perception of the school’s organisational climate.

Attitudes towards the teaching profession were measured using the 35-item Attitude towards the Teaching Profession Scale developed by Çetin (2006). The original instrument was developed with final-year pre-service teachers and comprises three dimensions—Love, Value, and Adaptation—rated on a five-point Likert-type scale ranging from 1 (strongly disagree) to 5 (strongly agree). Cronbach’s alpha for the full scale was reported as 0.95 in the scale-development study (Çetin, 2006). Use of the instrument with practising teachers is not without precedent. Pehlivan (2017), for example, administered the 35-item Çetin scale to 434 practising general high-school teachers and reported an alpha coefficient of 0.94, while a teacher-context adaptation was also undertaken by Göloğlu Demir (2016).

Because the participants in the present study were practising teachers rather than prospective teachers, wording that presupposed entry into a future profession was reviewed before data collection and, where necessary, reformulated to refer to teachers’ current professional experience. The revised wording was evaluated by two specialists in Turkish language and one specialist in educational administration for semantic equivalence, linguistic clarity, and contextual appropriateness. The revisions were restricted to temporal and role framing; the intended attitudinal content and the three-dimensional structure of the instrument were retained. Negatively worded items were reverse-coded before analysis. Love, Value, and Adaptation scores were calculated and used as indicators of the higher-order ATTP construct. In the present sample, internal consistency was acceptable for the full scale (α = 0.867) and for Love (α = 0.773), Value (α = 0.771), and Adaptation (α = 0.747). Higher scores indicate more favourable attitudes towards the teaching profession.

Teacher burnout was measured using the Maslach Burnout Inventory, developed by Maslach and Jackson (1981) and adapted into Turkish by Ergin (1992). The inventory comprises 22 items across three dimensions. Items are rated on a five-point frequency scale ranging from 0 (never) to 4 (always). In the Turkish adaptation study, Cronbach’s alpha coefficients were reported as 0.83 for emotional exhaustion, 0.65 for depersonalisation, and 0.72 for personal accomplishment (Ergin, 1992). Items requiring reverse scoring were recoded in accordance with the scoring instructions. Scores for the three subdimensions were calculated and used as indicators of the higher-order burnout construct. Higher scores indicate higher teacher burnout.

### 2.4 Ethical considerations

The study was conducted in accordance with the Declaration of Helsinki and was approved by the Ethics Committee of Cyprus Science University on 25 November 2024 (approval no. 2024/11.006). Participation was voluntary, and written informed consent was obtained from all participants before data collection.

### 2.5 Data analysis

Before the analyses, the dataset was examined for missing values, data-entry errors, and outlying observations. Means, standard deviations, skewness, and kurtosis were calculated for the study variables. Skewness and kurtosis values within acceptable limits were taken to indicate that the distributions were suitable for the subsequent analyses (Hair et al., 2019; Kline, 2023). Pearson correlation coefficients were used as a preliminary analysis of the bivariate associations among organisational climate, attitudes towards the teaching profession, and teacher burnout.

Because all study variables were obtained from the same participants using self-report instruments, the possibility of common-method variance was assessed using Harman’s single-factor test. All 96 measurement items were entered simultaneously into an unrotated single-factor principal component analysis. The first factor accounted for 11.621% of the total variance. As this proportion was substantially below the commonly used 50% threshold, the results did not suggest that a single factor accounted for most of the covariance among the measurement items. Common-method variance was therefore considered unlikely to constitute a major concern, although the diagnostic nature of Harman’s test was acknowledged (Podsakoff et al., 2003).

Potential multicollinearity among the predictor constructs was assessed using inner-model variance inflation factor (VIF) values. VIF values below 5.00 were taken to indicate the absence of problematic multicollinearity among the predictors (Hair et al., 2019).

The reliability and convergent validity of the reflective measurement models were assessed using standardised indicator loadings, Cronbach’s alpha, composite reliability (CR), and average variance extracted (AVE). Subdimension scores were used as indicators of the corresponding higher-order constructs. Cronbach’s alpha and CR values of 0.70 or above were regarded as evidence of acceptable internal consistency and construct reliability (Hair et al., 2019; Nunnally and Bernstein, 1994). An AVE value of 0.50 or above was regarded as evidence of convergent validity (Fornell and Larcker, 1981). Indicators with loadings between 0.40 and 0.70 were retained when CR and AVE remained acceptable and the indicators were considered theoretically relevant to their respective constructs (Hair et al., 2019). Consistent with this conceptualisation, organisational climate was specified as a reflective higher-order construct; its empirical coherence was examined through the higher-order measurement properties and the pattern of correlations among its six subdimensions.

Because the ATTP wording had been contextually adjusted for practising teachers, additional item-level checks were conducted for this instrument. Sampling adequacy and factorability were examined using the Kaiser-Meyer-Olkin (KMO) statistic and Bartlett’s test of sphericity. The number of factors supported by the item correlation matrix was additionally examined using parallel analysis. Internal consistency was calculated for the full ATTP scale and for each of its three subdimensions. These checks were used as sample-specific psychometric evidence and did not replace the higher-order measurement-model assessment used for hypothesis testing.

Discriminant validity was assessed using the heterotrait-monotrait ratio (HTMT) to determine whether the constructs were sufficiently distinct. HTMT values below 0.85 were regarded as evidence of empirical distinctiveness. In addition, 95% confidence intervals obtained from 5,000 bootstrap samples were examined, and discriminant validity was considered supported when the intervals did not include 1.00 (Henseler et al., 2015).

After the measurement model met the required reliability and validity criteria, the research hypotheses were tested using partial least squares structural equation modelling (PLS-SEM). The structural model specified paths from organisational climate to attitudes towards the teaching profession and teacher burnout, and from attitudes towards the teaching profession to teacher burnout. Age, educational qualification, tenure at the current school, and total teaching experience were also included as covariates for attitudes towards the teaching profession and burnout.

The statistical significance of the structural associations was evaluated using standardised path coefficients (β), standard errors, t values, *p* values, and bias-corrected 95% confidence intervals obtained from 5,000 bootstrap samples. The explained variance of the endogenous constructs was assessed using the coefficient of determination (R^2^) and adjusted R^2^. The individual contribution of each predictor to an endogenous construct was evaluated using the f^2^ effect-size coefficient.

The statistical mediating role of attitudes towards the teaching profession in the association between organisational climate and teacher burnout was tested using bootstrapping. An indirect association was regarded as statistically significant when its 95% confidence interval did not include zero (Hayes, 2022; Preacher and Hayes, 2008). A partial mediation pattern was inferred when the direct and indirect associations were both statistically significant; when they had the same direction, the pattern was classified as complementary partial mediation.

The out-of-sample predictive performance of the structural model was examined using the PLSpredict procedure with 10-fold cross-validation and 10 repetitions. Predictive-relevance coefficients (Q^2^predict) and prediction errors from the PLS-SEM model were compared with those from a linear-model benchmark. Q^2^predict values above zero and lower PLS-SEM prediction errors than the corresponding linear-model values were treated as evidence of out-of-sample predictive performance.

Three supplementary dimension-level analyses were conducted to examine the multidimensional structure of the study constructs more closely. First, Pearson correlations were calculated among the 12 subdimensions representing organisational climate, attitudes towards the teaching profession, and burnout. Second, the six organisational-climate dimensions were entered simultaneously into a standardised multiple-regression model predicting attitudes towards the teaching profession. Third, Love, Value, and Adaptation were entered simultaneously into a standardised multiple-regression model predicting overall burnout, together with overall organisational climate and the four background and career-related covariates. Variance inflation factors were examined before the dimension-specific coefficients were interpreted. These analyses were treated as supplementary to the higher-order PLS-SEM model.

Because the study used a cross-sectional design, the directional paths specified in the structural model were not interpreted as evidence of causality. The standardised path coefficients were treated as statistical associations defined by the theoretical model rather than as experimentally established causal effects (Kline, 2023).

## 3 Results

### 3.1 Descriptive statistics

Descriptive statistics, distributional indicators, and correlation coefficients for the study variables are presented in Table 2.

**Table 2:** Descriptive statistics, distributional indicators, and correlations for the study variables.

Constructs	Mean	SD	Skewness	Kurtosis	1	2	3
Organisational climate	3.41	0.220	−0.009	−0.451	1		
Attitudes towards the teaching profession	2.53	0.595	0.258	−0.499	0.357**	1	
Burnout	2.54	0.610	−0.477	0.048	−0.434**	−0.510**	1

M = mean; SD = standard deviation. **p < 0.01, two-tailed.

As shown in Table 2, the mean perceived organisational climate score was above the scale midpoint (M = 3.41, SD = 0.220). The mean scores for attitudes towards the teaching profession (M = 2.53, SD = 0.595) and burnout (M = 2.54, SD = 0.610) were close to their respective scale midpoints. Skewness values ranged from −0.477 to 0.258, whereas kurtosis values ranged from −0.499 to 0.048. These values indicated that no substantial univariate departure from normality was present (Hair et al., 2019; Kline, 2016). Organisational climate was positively correlated with attitudes towards the teaching profession (r = 0.357, *p* < 0.01) and negatively correlated with burnout (r = −0.434, *p* < 0.01). Attitudes towards the teaching profession were also negatively correlated with burnout (r = −0.510, *p* < 0.01). The directions of these correlations were consistent with the proposed research model.

### 3.2 Evaluating the measurement model

Following the descriptive and preliminary analyses, the measurement model was evaluated to assess the reliability and validity of the instruments. Standardised indicator loadings, Cronbach’s alpha, composite reliability, and average variance extracted were examined.

Table 3 presents the reliability and convergent-validity results for the study constructs. Cronbach’s alpha coefficients ranged from 0.845 to 0.867, indicating satisfactory internal consistency. Composite reliability values ranged from 0.840 to 0.880 and exceeded the recommended threshold of 0.70 (Hair et al., 2019). The AVE values ranged from 0.551 to 0.637 and were above the recommended threshold of 0.50, supporting convergent validity (Fornell and Larcker, 1981). Standardised subdimension loadings ranged from 0.685 to 0.825 and were considered acceptable (Hair et al., 2019). Overall, the results demonstrated adequate internal consistency, construct reliability, and convergent validity for organisational climate, attitudes towards the teaching profession, and burnout.

**Table 3:** Reliability and convergent validity results for the study constructs.

Construct	Cronbach’s alpha	CR	AVE	Indicator	Loading
Organisational climate	0.863	0.880	0.551	OC_D1	0.756
				OC_D2	0.749
				OC_D3	0.754
				OC_D4	0.685
				OC_D5	0.775
				OC_D6	0.731
Attitudes towards the teaching profession	0.867	0.841	0.637	ATTP_D1	0.794
				ATTP_D2	0.792
				ATTP_D3	0.809
Burnout	0.845	0.840	0.637	BO_D1	0.787
				BO_D2	0.781
				BO_D3	0.825

CR = composite reliability; AVE = average variance extracted. The indicators represent the subdimension scores of the corresponding higher-order constructs.

The additional item-level assessment of the ATTP instrument provided further sample-specific support for its use with practising teachers. The KMO value was 0.883, and Bartlett’s test of sphericity was statistically significant, χ^2^(595) = 2549.37, *p* < 0.001, indicating that the item correlation matrix was suitable for factor analysis. Parallel analysis supported the retention of the theoretically specified three-factor structure comprising Love, Value, and Adaptation. These findings complemented the satisfactory reliability and convergent-validity results obtained for ATTP in the higher-order measurement model.

Discriminant validity was assessed using the heterotrait-monotrait ratio (HTMT) to determine whether the constructs were empirically distinct. HTMT values were calculated using the subdimension scores as indicators of their corresponding general constructs. The results are presented in Table 4.

**Table 4:** Discriminant validity results based on the HTMT criterion.

Construct pair	HTMT	2.5% CI	97.5% CI
Organisational climate ↔ Attitudes towards the teaching profession	0.460	0.360	0.560
Organisational climate ↔ Burnout	0.556	0.463	0.644
Attitudes towards the teaching profession ↔ Burnout	0.701	0.611	0.788

HTMT, heterotrait-monotrait ratio; CI, confidence interval. HTMT values were calculated using the subdimension scores as construct indicators. Confidence intervals were obtained from 5,000 bootstrap samples.

As shown in Table 4, the HTMT values ranged from 0.460 to 0.701 and were below the recommended threshold of 0.85. Of particular relevance to the conceptual-overlap question, the HTMT value was 0.460 (95% CI [0.360, 0.560]) for organisational climate versus ATTP and 0.701 (95% CI [0.611, 0.788]) for ATTP versus burnout. Neither confidence interval included 1.00. These results indicate that professional attitudes shared meaningful variance with both constructs while remaining empirically distinguishable from perceptions of the school context and from burnout (Henseler et al., 2015).

### 3.3 Evaluation of the structural model

After the measurement model had satisfied the specified reliability and validity criteria, the structural associations were evaluated. Before the path coefficients were examined, potential multicollinearity among the predictor variables was assessed using variance inflation factor values. The results are presented in Table 5.

**Table 5:** Inner VIF values for the structural model.

Predictor variable	Attitudes towards the teaching profession	Burnout
Organisational climate	1.087	1.174
Attitudes towards the teaching profession	—	1.336
Age	1.046	1.070
Educational qualification	1.052	1.083
Years at the current school	1.049	1.073
Total teaching experience	1.071	1.159

VIF, variance inflation factor; —, no structural association was specified.

As shown in Table 5, the VIF values ranged from 1.046 to 1.336. All values were substantially below the recommended threshold of 5.00, indicating that problematic multicollinearity was not present among the predictor variables (Hair et al., 2019). The structural associations were therefore evaluated without evidence of a multicollinearity concern.

After no problematic multicollinearity was identified, the hypothesised associations among organisational climate, attitudes towards the teaching profession, and burnout were tested simultaneously using PLS-SEM. The structural-model results are presented in Figure 2, and the R^2^ and adjusted R^2^ values are reported in Table 6.

**Figure 2:**
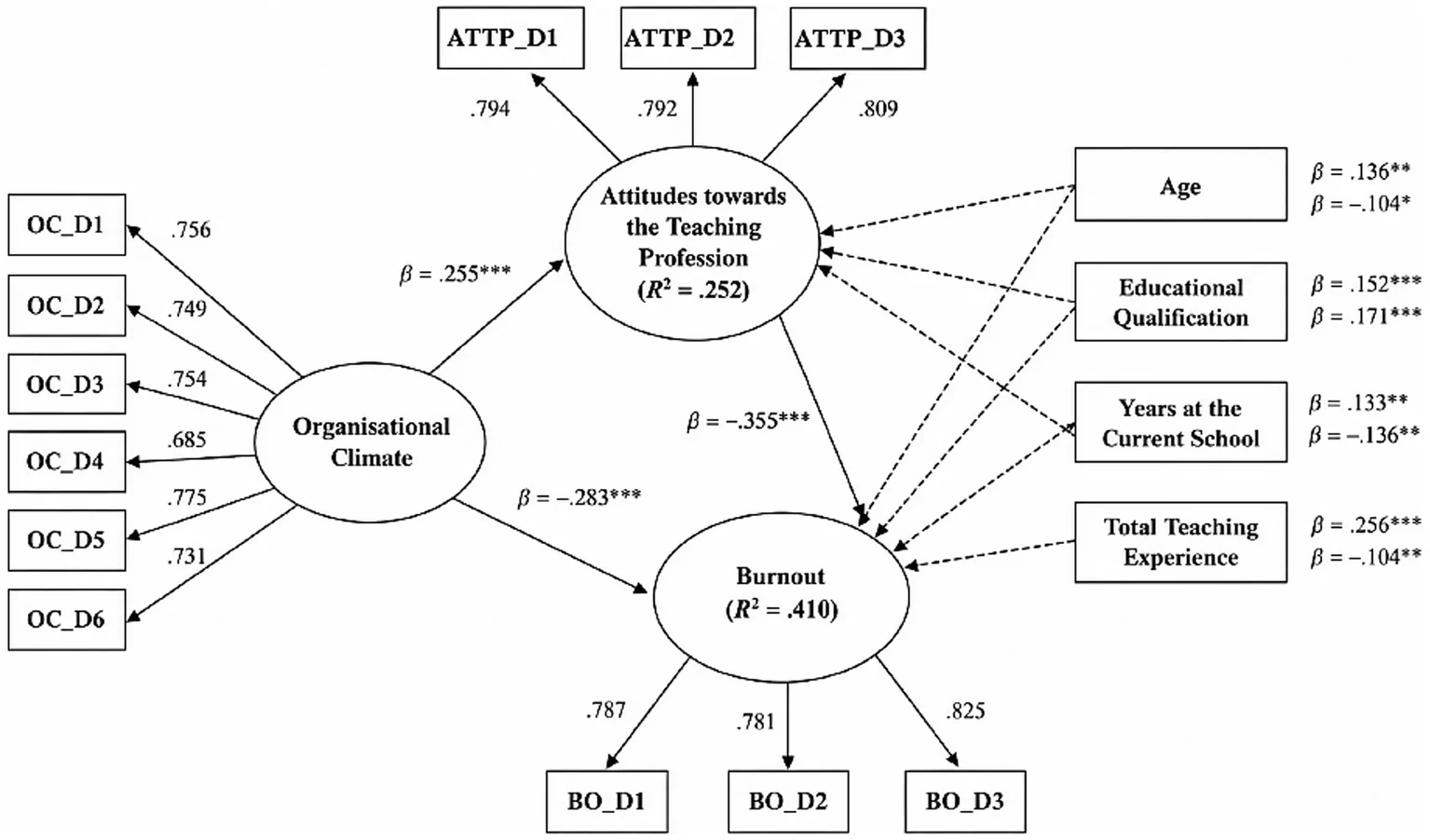
Results of the structural model.

**Table 6:** Explanatory power of the structural model.

Endogenous construct	R^2^	Adjusted R^2^
Attitudes towards the Teaching Profession	0.252	0.243
Burnout	0.410	0.402

R², coefficient of determination.

As shown in Table 6, the structural model explained 25.2% of the variance in attitudes towards the teaching profession (R^2^ = 0.252) and 41.0% of the variance in burnout (R^2^ = 0.410). The adjusted R^2^ values were 0.243 for attitudes towards the teaching profession and 0.402 for burnout. The small differences between the R^2^ and adjusted R^2^ values indicated that adjustment for the number of predictors had little influence on the estimated explanatory power of the model. A larger proportion of variance was explained in burnout than in attitudes towards the teaching profession.

After the explanatory power of the structural model was evaluated, the hypothesised direct associations were tested using 5,000 bootstrap samples. The results are presented in Table 7.

**Table 7:** Results of direct-association hypothesis testing.

Hypothesis	Structural path	β	Boot SE	t	*p*	95% CI	f^2^	Conclusion
H1	OC → ATTP	0.255	0.043	5.968	<0.001	[0.169, 0.337]	0.080	Supported
H2	OC → BO	−0.283	0.038	−7.444	<0.001	[−0.355, −0.208]	0.115	Supported
H3	ATTP → BO	−0.355	0.041	−8.593	<0.001	[−0.435, −0.273]	0.160	Supported

OC, organisational climate; ATTP, attitudes towards the teaching profession; BO, burnout; β, standardised association coefficient; Boot SE, bootstrap standard error; CI, confidence interval; f², effect size. Bootstrap resamples = 5,000.

As shown in Table 7, organisational climate was positively and statistically significantly associated with attitudes towards the teaching profession (β = 0.255, Boot SE = 0.043, t = 5.968, *p* < 0.001, 95% CI [0.169, 0.337]); therefore, H1 was supported. Organisational climate was negatively and statistically significantly associated with burnout (β = −0.283, Boot SE = 0.038, t = −7.444, *p* < 0.001, 95% CI [−0.355, −0.208]), supporting H2. Attitudes towards the teaching profession were also negatively and statistically significantly associated with burnout (β = −0.355, Boot SE = 0.041, t = −8.593, *p* < 0.001, 95% CI [−0.435, −0.273]), supporting H3. None of the bootstrap confidence intervals included zero. The f^2^ values indicated small effects for the associations of organisational climate with attitudes towards the teaching profession and burnout, whereas the association between attitudes towards the teaching profession and burnout produced the largest effect.

Following the analysis of the direct associations, the statistical mediating role of attitudes towards the teaching profession in the association between organisational climate and burnout was tested using bootstrapping. The direct, indirect, and total associations are presented in Table 8.

**Table 8:** Statistical mediation results for attitudes towards the teaching profession.

Hypothesis	Association	Structural path	β	Boot SE	t	*p*	95% CI	Conclusion
H4	Direct association	OC → BO	−0.283	0.038	−7.444	< 0.001	[−0.355, −0.208]	Statistically significant
	Indirect association	OC → ATTP → BO	−0.090	0.019	−4.810	< 0.001	[−0.130, −0.056]	H4 supported
	Total association	OC → BO	−0.373	0.039	−9.650	< 0.001	[−0.447, −0.297]	Statistically significant

OC, Organisational Climate; ATTP, Attitudes towards the Teaching Profession; BO, Burnout; β, standardised association coefficient; SE, bootstrap standard error; CI, confidence interval. The statistically significant direct and indirect associations had the same direction, indicating a complementary partial mediation pattern.

As shown in Table 8, the indirect association between organisational climate and burnout through attitudes towards the teaching profession was negative and statistically significant (β = −0.090, Boot SE = 0.019, t = −4.810, *p* < 0.001, 95% CI [−0.130, −0.056]). Because the bootstrap confidence interval did not include zero, H4 was supported. The direct association between organisational climate and burnout also remained negative and statistically significant after attitudes towards the teaching profession had been included in the model (β = −0.283, Boot SE = 0.038, t = −7.444, *p* < 0.001, 95% CI [−0.355, −0.208]). As both the direct and indirect associations were statistically significant and operated in the same direction, the findings indicated a complementary partial mediation pattern. The total association between organisational climate and burnout was likewise negative and statistically significant (β = −0.373, Boot SE = 0.039, t = −9.650, *p* < 0.001, 95% CI [−0.447, −0.297]).

After the hypothesised direct and indirect associations were evaluated, the associations of the covariates with attitudes towards the teaching profession and burnout were examined. The results are presented in Table 9.

**Table 9:** Associations of the background and career-related covariates.

Endogenous construct	Covariate	β	Boot SE	t	*p*	95% CI	f^2^
Attitudes towards the teaching profession	Age	0.136	0.042	3.205	0.001	[0.052, 0.219]	0.023
	Educational qualification	0.152	0.043	3.563	< 0.001	[0.067, 0.234]	0.029
	Years at the current school	0.133	0.043	3.125	0.002	[0.049, 0.217]	0.023
	Total teaching experience	0.256	0.042	6.075	< 0.001	[0.169, 0.337]	0.082
Burnout	Age	−0.104	0.041	−2.543	0.011	[−0.182, −0.024]	0.017
	Educational qualification	0.171	0.040	4.299	< 0.001	[0.093, 0.249]	0.046
	Years at the current school	−0.136	0.040	−3.444	0.001	[−0.215, −0.058]	0.029
	Total teaching experience	−0.104	0.038	−2.705	0.007	[−0.179, −0.028]	0.016

The covariate associations were not specified as formal hypotheses; therefore, hypothesis conclusions were not assigned.

As shown in Table 9, age, educational qualification, years at the current school, and total teaching experience were positively and statistically significantly associated with attitudes towards the teaching profession. The strongest association was observed for total teaching experience (β = 0.256, Boot SE = 0.042, t = 6.075, *p* < 0.001, 95% CI [0.169, 0.337]). Educational qualification (β = 0.152, Boot SE = 0.043, t = 3.563, *p* < 0.001, 95% CI [0.067, 0.234]), age (β = 0.136, Boot SE = 0.042, t = 3.205, *p* = 0.001, 95% CI [0.052, 0.219]), and years at the current school (β = 0.133, Boot SE = 0.043, t = 3.125, *p* = 0.002, 95% CI [0.049, 0.217]) were also positively associated with professional attitudes.

For burnout, age (β = −0.104, Boot SE = 0.041, t = −2.543, *p* = 0.011, 95% CI [−0.182, −0.024]), years at the current school (β = −0.136, Boot SE = 0.040, t = −3.444, *p* = 0.001, 95% CI [−0.215, −0.058]), and total teaching experience (β = −0.104, Boot SE = 0.038, t = −2.705, *p* = 0.007, 95% CI [−0.179, −0.028]) were negatively and statistically significantly associated with burnout. Educational qualification was positively and statistically significantly associated with burnout (β = 0.171, Boot SE = 0.040, t = 4.299, *p* < 0.001, 95% CI [0.093, 0.249]). None of the bootstrap confidence intervals included zero. The f^2^ values ranged from 0.016 to 0.082, indicating that the individual contributions of the covariates were generally small.

After the explanatory power and the direct and indirect associations of the structural model had been evaluated, its out-of-sample predictive performance was examined using the PLSpredict procedure. The results are presented in Table 10.

**Table 10:** Out-of-sample predictive performance of the structural model.

Endogenous construct	*n*	Q^2^predict range	Q^2^predict > 0	PLS RMSE < LM	PLS MAE < LM	Assessment
Attitudes towards the Teaching Profession	3	0.129–0.180	3/3	3/3	3/3	High
Burnout	3	0.174–0.291	3/3	3/3	3/3	High
Overall	6	0.129–0.291	6/6	6/6	6/6	High

*n*, number of indicators; Q²predict, predictive relevance; PLS, partial least squares model; LM, linear model benchmark; RMSE, root mean squared error; MAE, mean absolute error. Predictive performance was assessed using 10-fold cross-validation with 10 repetitions.

As shown in Table 10, the Q^2^predict values ranged from 0.129 to 0.180 for the subdimension indicators of attitudes towards the teaching profession and from 0.174 to 0.291 for the burnout indicators. All six indicators had Q^2^predict values above zero, indicating predictive relevance for observations omitted during cross-validation. In addition, the PLS-SEM model produced lower RMSE and MAE values than the corresponding linear-model benchmarks for all indicators. These findings indicated high out-of-sample predictive performance for both attitudes towards the teaching profession and burnout (Shmueli et al., 2019).

### 3.4 Supplementary dimension-level analyses

Pearson correlations among the 12 subdimensions of organisational climate, attitudes towards the teaching profession, and burnout were examined to provide a more detailed assessment of their dimension-level associations. The results are presented in Table 11.

**Table 11:** Pearson correlations among the 12 subdimensions of the study constructs.

Subdimension	1	2	3	4	5	6	7	8	9	10	11	12
1. Supportive principal behaviour	1											
2. Directive principal behaviour	−0.454	1										
3. Restrictive principal behaviour	−0.467	0.482	1									
4. Intimate teacher behaviour	0.458	−0.420	−0.441	1								
5. Collegial teacher behaviour	0.504	−0.500	−0.518	0.399	1							
6. Disengaged teacher behaviour	−0.474	0.471	0.438	−0.374	−0.501	1						
7. Love	0.219	−0.227	−0.199	0.151	0.238	−0.164	1					
8. Value	0.178	−0.269	−0.238	0.194	0.202	−0.177	0.437	1				
9. Adaptation	0.209	−0.270	−0.223	0.223	0.210	−0.204	0.468	0.464	1			
10. Emotional exhaustion	−0.237	0.314	0.279	−0.191	−0.272	0.253	−0.324	−0.295	−0.381	1		
11. Depersonalisation	−0.233	0.310	0.258	−0.177	−0.217	0.201	−0.262	−0.255	−0.245	0.407	1	
12. Reduced personal accomplishment	−0.288	0.312	0.287	−0.266	−0.266	0.221	−0.356	−0.389	−0.368	0.484	0.473	1

Pearson correlation coefficients are reported for the 12 subdimensions. All correlations were statistically significant at *p* < 0.01 (two-tailed).

As shown in Table 11, the absolute correlations among the six organisational-climate dimensions ranged from |r| = 0.374 to 0.518 (all *p* < 0.01). The three ATTP dimensions were moderately intercorrelated (Love–Value r = 0.437, Love–Adaptation r = 0.468, and Value–Adaptation r = 0.464), indicating related but non-redundant facets. Their correlations with overall organisational climate were 0.270 for Love, 0.285 for Value, and 0.302 for Adaptation, whereas their correlations with overall burnout were −0.398, −0.397, and −0.427, respectively. At the more specific subdimension level, no ATTP-burnout correlation exceeded |r| = 0.389; for Adaptation, correlations were −0.381 with emotional exhaustion, −0.245 with depersonalisation, and −0.368 with reduced personal accomplishment. Across all constructs, no cross-construct subdimension correlation exceeded |r| = 0.389. This pattern is consistent with substantive association rather than empirical redundancy and provides additional evidence that the Adaptation factor, although related to burnout, was not functioning as a proxy for a burnout dimension.

A supplementary dimension-level regression was then conducted to determine which organisational-climate dimension showed the strongest unique association with attitudes towards the teaching profession. The six climate dimensions were entered simultaneously, together with the four background and career-related covariates. The results are presented in Table 12.

**Table 12:** Supplementary dimension-level associations of organisational climate with attitudes towards the teaching profession.

Dimension	β	SE	t	*p*	95% CI	f^2^
Supportive principal behaviour	0.051	0.055	0.929	0.353	[−0.057, 0.159]	0.002
Directive principal behaviour	−0.126	0.055	−2.296	0.022	[−0.234, −0.018]	0.013
Restrictive principal behaviour	−0.048	0.055	−0.875	0.382	[−0.156, 0.060]	0.002
Intimate teacher behaviour	0.056	0.051	1.096	0.274	[−0.045, 0.157]	0.003
Collegial teacher behaviour	0.081	0.056	1.442	0.150	[−0.029, 0.192]	0.005
Disengaged teacher behaviour	0.023	0.054	0.424	0.672	[−0.083, 0.128]	<0.001

β, standardised regression coefficient; SE, standard error; CI, confidence interval; f², local effect size. Age, educational qualification, tenure at the current school, and total teaching experience were included as covariates but are omitted from the table for brevity.

The supplementary dimension-level model was statistically significant, F(10, 413) = 14.29, *p* < 0.001, and explained 25.7% of the variance in attitudes towards the teaching profession (R^2^ = 0.257; adjusted R^2^ = 0.239). VIF values for the six organisational-climate dimensions ranged from 1.46 to 1.76, indicating no problematic multicollinearity. Directive principal behaviour showed the largest unique climate-dimension coefficient and was the only organisational-climate dimension with a statistically significant unique association with professional attitudes (β = −0.126, SE = 0.055, t = −2.296, *p* = 0.022, 95% CI [−0.234, −0.018], f^2^ = 0.013). The remaining dimension-specific coefficients were small and statistically non-significant. Because this analysis was supplementary and *post hoc*, the dimension-level pattern was interpreted as exploratory rather than as a replacement for the higher-order structural model.

A second supplementary regression was conducted to determine whether the association between ATTP and burnout was concentrated in a single ATTP dimension. Love, Value, and Adaptation were entered simultaneously together with overall organisational climate and the four covariates. The model was statistically significant, F(8, 415) = 36.23, *p* < 0.001, and explained 41.1% of the variance in burnout (R^2^ = 0.411; adjusted R^2^ = 0.400); the largest VIF was 1.54. All three ATTP dimensions retained statistically significant negative associations with burnout: Love, β = −0.151, SE = 0.045, t = −3.337, *p* = 0.001, 95% CI [−0.239, −0.062]; Value, β = −0.126, SE = 0.045, t = −2.799, *p* = 0.005, 95% CI [−0.215, −0.038]; and Adaptation, β = −0.170, SE = 0.047, t = −3.626, *p* < 0.001, 95% CI [−0.262, −0.078]. This exploratory analysis indicates that the H3 association was not attributable solely to the Adaptation factor or to a single attitudinal facet.

## 4 Discussion

This study examined the direct and indirect associations among organisational climate, attitudes towards the teaching profession, and teacher burnout. A more positive perception of organisational climate was associated with more favourable attitudes towards the teaching profession and lower burnout. More favourable professional attitudes were also associated with lower burnout. In addition, the results supported a complementary partial mediation pattern in the indirect association between organisational climate and burnout through attitudes towards the teaching profession. These findings indicate that teachers’ professional and psychological experiences should be considered not only in relation to individual characteristics but also alongside the social and organisational conditions of the schools in which they work (Bakker et al., 2023; Grayson and Alvarez, 2008).

The positive association between organisational climate and attitudes towards the teaching profession supported H1. This finding indicates that teachers’ evaluations of their profession are connected not only to personal preferences or predispositions formed before entering teaching but also to the managerial practices and social relationships experienced at school (Skaalvik and Skaalvik, 2011; Toropova et al., 2021). Organisational conditions such as administrator support, collegial collaboration, mutual trust, autonomy, and participation in decision-making have been associated with teachers feeling valued and supported (Collie et al., 2012; Skaalvik and Skaalvik, 2018; Toropova et al., 2021). Positive experiences of the work environment may therefore co-occur with evaluations of teaching as more meaningful and valuable. Previous studies reporting positive associations between school climate and attitudes towards the teaching profession are consistent with the present finding (Sezer and Çevik, 2023). The direction of this association is consistent with Sezer and Çevik (2023), although their evidence was obtained from pre-school teachers rather than practising lower-secondary and high-school teachers. The present finding therefore extends, rather than directly replicates, that association across a different educational level and after background and career-related covariates were taken into account.

This association can be considered within the motivational process described by Job Demands-Resources theory. Job resources facilitate task completion and are associated with favourable motivational outcomes (Bakker and Demerouti, 2017; Bakker et al., 2023). Organisational climate does not represent a single job resource; rather, it reflects teachers’ perceptions of the overall quality of managerial and social resources within the school (Schneider et al., 2013). The association between a positive organisational climate and favourable professional attitudes may therefore suggest that supportive working conditions are connected to the meaning teachers assign to their profession (Toropova et al., 2021; van Beurden et al., 2017). Given the cross-sectional design, however, this association should not be interpreted as evidence that organisational climate improves professional attitudes over time.

The supplementary dimension-level analysis further indicated that the organisational-climate dimensions were not equivalent in their unique associations with professional attitudes. After the shared variance among the six climate dimensions and the background and career-related covariates had been taken into account, directive principal behaviour showed the strongest unique association with attitudes towards the teaching profession. Greater directive behaviour was associated with less favourable professional attitudes, whereas the unique coefficients for the remaining dimensions were small and statistically non-significant. This pattern is consistent with conceptual accounts in which excessive control and close supervision may be associated with less favourable evaluations of teachers’ professional roles (Hoy and Tarter, 1997; Skaalvik and Skaalvik, 2011; Yılmaz and Altınkurt, 2013). Because the analysis was supplementary, this dimension-specific finding should be interpreted cautiously and examined in future studies designed explicitly to compare organisational-climate dimensions.

The negative association between organisational climate and teacher burnout supported H2. This result is consistent with previous research linking the managerial and relational characteristics of schools to teacher burnout (Grayson and Alvarez, 2008; Malinen and Savolainen, 2016). Grayson and Alvarez (2008) reported associations between dimensions of school climate related to administration, student behaviour, and the school environment and different dimensions of burnout. Malinen and Savolainen (2016) identified both a direct association between perceived school climate and teacher burnout and an indirect association through teacher self-efficacy. More recent research has similarly reported that supportive and inclusive school climates are associated with lower teacher burnout (Postigo-Zegarra et al., 2025; Yang and Zhou, 2025). Recent studies provide convergent evidence while identifying different psychological processes. Yang and Zhou (2025) reported a negative association between inclusive school climate and burnout with teaching efficacy as a partial mediator, while Postigo-Zegarra et al. (2025) highlighted the interplay between school climate, emotional intelligence, and burnout. Harrison et al. (2025) similarly found that school climate was related to teacher wellbeing through basic psychological need satisfaction. The present study is consistent with the broader contextual pattern but differs in focusing on professional attitudes as a profession-specific evaluative mechanism.

At the same time, the association between organisational climate and burnout should not be understood as universal or invariant across educational contexts or teacher outcomes. Brestovanský and Sopoliga (2026), for example, found that burnout components did not moderate the relationship between school climate and reflective culture or the relationship between reflective culture and teachers’ perceptions of the curriculum. Although their model addressed different outcomes and tested moderation rather than the direct climate-burnout association examined here, their findings show that burnout does not necessarily alter every pathway through which school climate is connected to teachers’ professional functioning. The magnitude and role of organisational climate may therefore depend on the specific outcome, organisational process, and educational context under consideration. Accordingly, the present H2 finding should be interpreted as evidence of a negative association in this sample rather than as a universal relationship assumed to operate identically across settings.

According to Job Demands-Resources theory, resources such as administrator support, trust, autonomy, and collegial collaboration may be inversely associated with the psychological costs of teachers’ job demands (Bakker and Demerouti, 2017; Bakker et al., 2023). Insufficient support, adverse interpersonal relationships, and incongruence between teachers’ values and school practices may instead co-occur with the depletion of emotional resources (Mijakoski et al., 2022; Skaalvik and Skaalvik, 2011). Teacher burnout therefore cannot be adequately understood solely through individual coping capacities or personal resilience (Agyapong et al., 2022; Mijakoski et al., 2022). The present finding supports considering burnout as an occupational well-being concern associated with leadership practices, work relationships, and social-organisational resources within schools.

The negative association between attitudes towards the teaching profession and burnout supported H3. Regarding teaching as meaningful and valuable has been associated with embracing the teaching role and willingly maintaining professional responsibilities (Andronache et al., 2014; Pekmezci and Dağdeviren Ertaş, 2024). Unfavourable evaluations of the profession may instead co-occur with loss of motivation, psychological distancing, and a weaker desire to remain in teaching (Skaalvik and Skaalvik, 2011). The association between more favourable professional attitudes and lower burnout therefore suggests that the meaning and value teachers assign to their profession are connected to their occupational well-being.

The present finding indicates that evidence concerning the association between professional attitudes and burnout, obtained predominantly among pre-service teachers, is also observable among practising lower-secondary and high-school teachers. Studies of pre-service teachers have reported that more favourable professional attitudes are associated with lower burnout and that teaching motivation may play an explanatory role in this association (Dikmen et al., 2023). Research among practising teachers has likewise linked teaching motivation, intentions to remain in the profession, and burnout (Skaalvik and Skaalvik, 2020). Favourable professional orientations such as professional identity and career calling have also been associated with lower burnout through work engagement and psychological capital (Zhang et al., 2024; Zhao et al., 2022). Although these constructs are conceptually distinct from attitudes towards the teaching profession, they collectively underscore the relevance of the meaning and value teachers assign to their profession for understanding burnout. The inverse association observed here is directly consistent with Şen and Koçak (2022) among practising physical education teachers and with Dikmen et al. (2023) among pre-service teachers. However, the present sample comprised practising lower-secondary and high-school teachers across subject areas, which reduces the extent to which the observed pattern can be attributed to a single discipline or to a pre-service context.

The supplementary psychometric and dimension-level findings are important for interpreting this association. Although some ATTP items contain affective language that may appear superficially close to burnout-related content, the empirical results did not support treating ATTP as interchangeable with burnout or organisational climate. The ATTP-burnout HTMT value was 0.701 and the ATTP-organisational-climate value was 0.460, with both bootstrap confidence intervals remaining below 1.00. Moreover, no correlation between an ATTP subdimension and a burnout subdimension exceeded |r| = 0.389, and Love, Value, and Adaptation each retained a unique negative association with burnout when entered simultaneously with organisational climate and the covariates. Adaptation therefore contributed to the association, but did not account for it on its own. These results reinforce the conceptual distinction between evaluating the teaching profession and experiencing occupational exhaustion, depersonalisation, or reduced personal accomplishment.

The statistical mediating role of attitudes towards the teaching profession in the indirect association between organisational climate and burnout supported H4. This finding indicates that the organisational conditions teachers experience at school are connected to their cognitive and affective evaluations of the profession and that these evaluations are, in turn, associated with burnout. Organisational conditions such as support, trust, collaboration, recognition, autonomy, and value congruence have been associated with more favourable evaluations of professional roles (Toropova et al., 2021; van Beurden et al., 2017). Favourable professional orientations have also been linked to stronger work engagement and lower burnout (Zhang et al., 2024; Zhao et al., 2022). The mediation pattern therefore suggests that organisational and psychological processes are interconnected in relation to teachers’ occupational well-being.

The mediation result can be interpreted with reference to the motivational process described by Job Demands-Resources theory. The theory proposes that job resources may support employee motivation and psychological connection to work and may also reduce the adverse consequences associated with job demands (Bakker and Demerouti, 2017; Bakker et al., 2023). In the present study, however, neither specific job demands nor discrete job resources were modelled separately; the mediation pattern should therefore not be interpreted as a direct empirical test of the JD-R motivational or health-impairment processes. Rather, the finding that a more positive organisational climate was associated with more favourable professional evaluations, which were in turn associated with lower burnout, suggests that teachers’ perceptions of a social-organisational context characterised by resource-relevant conditions are statistically connected to their profession-specific psychological orientations and occupational well-being. Attitudes towards the teaching profession may therefore be positioned as a profession-specific social-psychological mechanism that statistically accounts for part of the association between perceived organisational climate and burnout. This indirect association represents a statistical mediation pattern derived from cross-sectional data and should not be interpreted as evidence of a temporal causal process.

The direct association between organisational climate and burnout remained statistically significant in the mediation model, indicating that attitudes towards the teaching profession did not fully account for this association. The direct and indirect associations were statistically significant and had the same direction, a pattern consistent with complementary partial mediation. Organisational climate may be associated with burnout through several additional processes, including administrator support, workload organisation, collegial relationships, autonomy, and organisational trust (Grayson and Alvarez, 2008; Mijakoski et al., 2022). Attitudes towards the teaching profession should therefore be understood as one explanatory mechanism within the multidimensional association between organisational climate and teacher burnout.

The mediation finding should also be considered alongside recent studies that have identified other intervening processes between school context and teacher well-being. Teaching efficacy (Yang and Zhou, 2025), basic psychological need satisfaction (Harrison et al., 2025), emotion regulation (Li, 2025), and teacher self-efficacy (Malinen and Savolainen, 2016) have each been shown to account for part of the association between school conditions and teacher outcomes. The present result is consistent with this broader process-oriented literature in showing that contextual conditions and psychological functioning are linked indirectly as well as directly, but it differs in identifying teachers’ evaluation of the profession itself as the intervening construct. This distinction supports the interpretation of professional attitudes as one complementary mechanism rather than as a substitute for efficacy, need satisfaction, emotion regulation, or other established pathways.

This distinction is particularly important when the present findings are compared with efficacy-based mediation models. Lim and Eo (2014) showed that collective teacher efficacy mediated associations between dimensions of school organisational climate and teacher burnout, whereas Malinen and Savolainen (2016) identified teacher efficacy in behaviour management as an indirect pathway linking perceived school climate with burnout. Junça-Silva and Freire (2022) further demonstrated that work-family conflict can mediate associations between particular organisational-climate dimensions and burnout. The present mediator differs from these mechanisms because it does not represent a belief about individual or collective capability, nor a conflict between work and family roles; instead, it represents teachers’ cognitive and affective evaluation of the teaching profession itself. The current findings therefore complement rather than reproduce efficacy-based explanations by suggesting that organisational conditions may be associated with burnout not only through beliefs about what teachers can accomplish, but also through how they value, interpret, and regard the sustainability of teaching as a profession.

The findings for the background and career-related covariates indicated that age, educational qualification, tenure at the current school, and total teaching experience were positively associated with attitudes towards the teaching profession (see Table 9). The largest association was observed for total teaching experience, suggesting that time spent in the profession may be a distinguishing characteristic in teachers’ evaluations of their occupational roles. Although greater experience may be accompanied by more established appraisals of the demands, responsibilities, and meaning of teaching, this interpretation is supported only indirectly by studies reporting differences in professional attitudes across experience groups (Kuyumcu and Kaya, 2020; Mirze and Yılmaz, 2022). Previous findings concerning age, experience, and professional attitudes vary in direction and magnitude according to sample characteristics, educational level, and occupational context (Kuyumcu and Kaya, 2020; Mirze and Yılmaz, 2022). The present result should therefore not be interpreted as indicating that experience necessarily improves attitudes towards teaching; rather, it suggests that career stage is associated with teachers’ cognitive and affective evaluations of their profession (Zhou et al., 2024).

Age, tenure at the current school, and total teaching experience were negatively associated with burnout (see Table 9). This pattern is consistent with recent findings indicating higher burnout among younger and less experienced teachers (Cortez Soto and Heredia Escorza, 2026). It should not, however, be interpreted as evidence that age or accumulated professional experience is generally protective against burnout. In contrast to the present pattern, Alghamdi and Sideridis (2025), using latent burnout profiles among elementary and secondary teachers, reported that age and teaching experience positively predicted membership in higher-burnout profiles, with older and more experienced teachers showing greater burnout risk. The broader literature is similarly inconsistent, with some studies reporting greater burnout among younger and less experienced teachers and others identifying higher burnout among more experienced teachers (Nguyen and Kremer, 2022; Saloviita and Pakarinen, 2021; Yorulmaz and Altınkurt, 2018). These divergent findings suggest that the direction and magnitude of age- and experience-related associations may vary according to educational system, sample composition, career-stage distribution, working conditions, and analytic approach. The negative coefficients observed here should therefore be understood as sample-specific associations rather than as evidence that burnout necessarily declines with age or professional experience.

The positive associations of educational qualification with both attitudes towards the teaching profession and burnout represent a noteworthy pattern, suggesting that favourable professional orientation and psychological strain are not simple opposites (see Table 9). Teachers may regard their profession as meaningful and valuable while still experiencing burnout under demanding or unfavourable organisational conditions (Bakker and Demerouti, 2017; Zhou et al., 2024). The positive association between educational qualification and burnout is consistent with some research reporting higher burnout among teachers with higher educational qualifications (Sadeghi and Khezrlou, 2014). Meta-analytic evidence, however, indicates that the overall association of educational qualification with teacher burnout is very small and varies across research contexts (Yorulmaz and Altınkurt, 2018). One possible explanation is that teachers with higher qualifications may hold stronger expectations concerning career development, professional autonomy, recognition, or the use of their expertise, although this interpretation is a theoretical inference based on previous findings concerning value fit and career opportunities (van Beurden et al., 2017). Because career expectations and person-role fit were not measured directly, the positive association between educational qualification and burnout should be treated as a context-sensitive, exploratory finding.

The generally small f^2^ values for the covariates indicate that age, educational qualification, and professional experience were associated with teachers’ professional attitudes and burnout but were not dominant correlates of these outcomes (see Table 9). This interpretation is consistent with meta-analytic findings showing that demographic characteristics generally have very small or small associations with teacher burnout (Yorulmaz and Altınkurt, 2018). The broader literature on teacher well-being also indicates that work climate, meaningful work, social relationships, autonomous motivation, and psychological resources tend to show stronger associations than demographic characteristics (Zhou et al., 2024). The covariate results should therefore not be interpreted as deterministic profiles indicating that particular age, qualification, or experience groups are inevitably vulnerable to burnout (Zhou et al., 2024; Yorulmaz and Altınkurt, 2018). Because no directional hypotheses were specified for these variables, their associations should be regarded as secondary and explanatory findings that complement the focal model (Becker, 2005). Taken together, the results suggest that teachers’ professional and psychological experiences vary across career stages but cannot be understood independently of the social and organisational conditions of schools (Melzak et al., 2025; Zhou et al., 2024).

## 5 Implications

### 5.1 Academic implications

This study examined organisational climate, attitudes towards the teaching profession, and teacher burnout within the same structural model. The positive association between organisational climate and professional attitudes, the negative associations of organisational climate and professional attitudes with burnout, and the complementary partial mediation pattern collectively indicate that teachers’ psychological experiences need to be considered alongside their social-organisational work environments. The statistical mediation result suggests that part of the association between organisational climate and burnout is connected to the meaning and value teachers assign to their profession. This pattern is consistent with the account offered by Job Demands-Resources theory concerning the relationships among organisational resources, motivational orientations, and burnout (Bakker and Demerouti, 2017; Bakker et al., 2023).

The principal academic contribution of the study is the positioning of attitudes towards the teaching profession as a profession-specific cognitive and affective mechanism within the association between organisational climate and burnout. Whereas previous research has focused on constructs such as self-efficacy, work engagement, psychological capital, and professional identity, the present findings indicate that teachers’ evaluations of the meaning and value of their profession also contribute to explaining this association (Malinen and Savolainen, 2016; Zhang et al., 2024). The continuing statistical significance of the direct association further indicates that professional attitudes are not the only explanatory mechanism and that teacher burnout needs to be considered within a multidimensional organisational and psychological framework.

### 5.2 Practical implications

The direct association between organisational climate and burnout indicates that initiatives intended to support teacher well-being should not be limited to individual stress-management or personal coping programmes. School-level characteristics such as administrator support, trust, open communication, collegial collaboration, autonomy, and participation in decision-making may be monitored through regular and anonymous teacher feedback. Such feedback may be reviewed at the school level to identify areas requiring attention, including communication difficulties, perceived lack of support, and limited teacher participation in decision-making, and to inform context-specific improvement plans.

The partial mediation pattern also points to the relevance of practices that support teachers in experiencing their professional roles as meaningful and valued. Making teachers’ contributions visible, consulting them in decision-making, aligning professional-development opportunities with identified needs, and recognising achievements fairly may be considered in this regard. These practices should not, however, shift responsibility onto teachers to adopt more positive attitudes. Favourable professional evaluations are more appropriately supported alongside trustworthy and supportive organisational conditions.

### 5.3 Limitations and future research

The cross-sectional design precludes firm conclusions about temporal ordering or causal direction. The mediation result should therefore be interpreted as statistical support for a theoretically proposed indirect association rather than evidence of a causal mechanism. Longitudinal or time-lagged designs could examine whether changes in organisational climate are associated with subsequent changes in professional attitudes and burnout. In addition, all focal constructs were measured using self-report instruments; consequently, the findings cannot be assumed to be entirely independent of common-method influences. Future studies could incorporate school records, absence data, or assessments from additional sources.

Although geographical regions were considered during sampling, participants within each region were recruited on the basis of accessibility and voluntary participation, limiting the generalisability of the findings to all lower-secondary and high-school teachers in the TRNC. Replicating the model in other educational levels, school types, and educational systems could help assess the consistency of the associations across contexts. Organisational climate, attitudes towards the teaching profession, and burnout were modelled as higher-order constructs represented by their respective subdimensions. Although the supplementary analyses examined the unique associations of the organisational-climate dimensions with attitudes towards the teaching profession, a comprehensive dimension-level structural model incorporating the individual dimensions of all three constructs was not estimated. Future research could examine these more specific pathways and employ multilevel analyses to account for the clustering of teachers within schools. Because population-level data on the educational-qualification distribution of TRNC lower-secondary and high-school teachers were unavailable, the representativeness of the sample with respect to postgraduate qualifications could not be established. The 46.9% postgraduate proportion should therefore be considered when generalising the results, but it should not be assumed to be atypical solely on the basis of comparisons with other education systems, where qualification structures vary substantially (OECD, 2025).

The original ATTP instrument was developed with final-year pre-service teachers, and its use with practising teachers therefore required contextual attention. In the present study, future-oriented wording was reformulated where necessary, and the revised wording was reviewed by two Turkish-language specialists and one educational-administration specialist before administration. Prior use of the instrument with practising teachers and the sample-specific evidence obtained here—acceptable internal consistency, a three-factor solution supported by parallel analysis, coherent higher-order loadings, satisfactory discriminant validity, and non-redundant factor-level associations—support the present operationalisation. Nevertheless, contextual wording adaptation cannot by itself establish invariance across pre-service and practising-teacher populations. Future research should independently cross-validate the adapted wording in additional practising-teacher samples and, where feasible, compare it with instruments developed specifically for in-service professional attitudes.

A further limitation concerns the use of JD-R theory as an interpretative framework without direct measurement of specific job demands and discrete job resources. The present data therefore do not allow particular demands to be identified as sources of burnout, their relative importance to be compared, or the unique contribution of individual resources to organisational-climate perceptions to be estimated. Future research could assess job demands such as workload, time pressure, role conflict, and emotional demands alongside resource variables such as administrator support, autonomy, collegial support, and participation in decision-making. Modelling these variables together with organisational climate would permit a more direct test of JD-R processes and would help clarify whether climate operates primarily as a psychosocial appraisal of underlying organisational conditions, as a distinct contextual construct, or through both pathways.

## 6 Conclusion

This study examined the direct and indirect associations among lower-secondary and high-school teachers’ perceptions of organisational climate, their attitudes towards the teaching profession, and burnout in the TRNC. A more positive perception of organisational climate was associated with more favourable professional attitudes and lower burnout. More favourable attitudes towards the teaching profession were also associated with lower burnout.

The principal finding was the complementary partial mediation pattern involving attitudes towards the teaching profession in the association between organisational climate and burnout. This result indicates that the social and organisational conditions teachers experience at school are connected to their cognitive and affective evaluations of the profession and that these evaluations are, in turn, associated with burnout. The continuing statistical significance of the direct association between organisational climate and burnout indicates that professional attitudes do not fully account for this relationship and that teacher burnout should be considered as a multidimensional phenomenon.

The findings indicate that teacher burnout cannot be understood solely through individual characteristics or personal coping capacities. As an outcome that threatens occupational well-being, burnout should be considered within an integrated framework combining the social-organisational work environment of schools with the meaning and value teachers assign to their profession. The observed associations provide context-sensitive evidence from the TRNC concerning the interconnection between organisational dynamics and profession-specific psychological orientations in teacher well-being. Given the cross-sectional design, however, these associations should be interpreted as theoretically grounded statistical patterns rather than causal effects.

## Data Availability

The raw data supporting the conclusions of this article will be made available by the authors, without undue reservation.
